# Antimicrobial Properties of Freeze-Dried Hyaluronic Acid Sheets Containing Persimmon Tannin

**DOI:** 10.3390/ijms27156880

**Published:** 2026-08-01

**Authors:** Yasuhisa Sawai, Miho Hasumoto, Takanobu Takata, Arina Nakamichi, Mahmoud Gallab, Kunio Yoneto, Chika Yoneto, Satoshi Wada, Eiji Mitate, Seiji Yamaguchi, Hiroyuki Nakano

**Affiliations:** 1 Department of Oral and Maxillofacial Surgery, Faculty of Medicine, Kanazawa Medical University, Kahoku 920-0293, Japan; y-sawai@kanazawa-med.ac.jp (Y.S.); honjo@kanazawa-med.ac.jp (M.H.); michi-i@kanazawa-med.ac.jp (A.N.); wada-s@kanazawa-med.ac.jp (S.W.);; 2 Department of Pathology II, Faculty of Medicine, Kanazawa Medical University, Kahoku 920-0293, Japan; 3 Department of Pharmacy, Kanazawa Medical University Hospital, Kahoku 920-0293, Japan; 4 Department of Biomedical Sciences, College of Life and Health Sciences, Chubu University, Kasugai 487-0027, Japan; mahmoud@isc.chubu.ac.jp (M.G.); sy-esi@isc.chubu.ac.jp (S.Y.); 5 Ritapharma, Co., Ltd., Kyoto 600-8813, Japan; kyoneto04@gmail.com (K.Y.); c-yoneto@ritapharma.co.jp (C.Y.)

**Keywords:** antibacterial activity, hyaluronic acid, oral mucositis, persimmon tannin

## Abstract

Oral mucositis is a frequent adverse effect of cancer therapy and is often complicated by pain, oral dryness, and secondary infection. Freeze-dried hyaluronic acid (HA) sheets can protect and moisturize the oral mucosa, but their intrinsic antibacterial activity is limited. We developed freeze-dried HA sheets containing the commercial persimmon tannin product PANCIL^®^ PS-M and evaluated their release behavior, pH profile, antimicrobial activity, and in vitro cytocompatibility. Persimmon tannin release into phosphate-buffered saline was quantified over 8 h using the Folin–Ciocalteu method. Antimicrobial activity against *Streptococcus mitis*, *Prevotella intermedia*, and *Candida albicans* was assessed by colony counting, and cytocompatibility was evaluated using the human oral cell line T3M-1. Approximately 95% of the incorporated persimmon tannin was released within 8 h, while the surrounding pH remained near neutral to mildly alkaline. The antibacterial HA sheets markedly reduced viable counts of *S. mitis* and *P. intermedia*, whereas no inhibitory effect was observed against *C. albicans*. Neither HA sheet nor antibacterial HA sheet solutions significantly reduced T3M-1 cell viability at concentrations up to 100 µg/mL after 24 h. These findings suggest that persimmon tannin-containing HA sheets may provide sustained antibacterial activity without detectable cytotoxicity under the tested conditions and may serve as a topical adjunct for cancer treatment-related oral mucositis.

## 1. Introduction

Oral mucositis is a common adverse event associated with chemotherapy and radiotherapy. It has been reported to occur in 30–40% of patients receiving anticancer drugs, 70–90% of patients undergoing hematopoietic stem cell transplantation, and almost 100% of those treated with a combination of radiotherapy and chemotherapy [1]. Oral mucositis is sometimes accompanied by ulceration, bleeding, and pain, leading to reduced oral intake and malnutrition, thereby lowering quality of life (QOL) and potentially making continuation of cancer treatment difficult. The severity of oral mucositis has been reported to correlate with the degree of oral dryness and the abundance of oral *Candida* species [2]. Clinical oral fungal infections are common during cancer therapy, with a systematic review estimating a weighted prevalence of approximately 39.1% during treatment [3]. In profoundly immunosuppressed settings, such as hematopoietic stem cell transplantation, bacterial bloodstream infections have been reported to occur in approximately 5–10% of autologous and 20–30% of allogeneic recipients [4]. In addition, ulcers associated with oral mucositis may serve as a portal of entry for oral bacteria, which can cause bacteremia and sepsis. Among oral bacteria, *Streptococcus mitis* is a predominant oral commensal streptococcus and has been implicated in bloodstream infections in patients with cancer and neutropenia [5]. *Prevotella intermedia* is an anaerobic bacterium associated with oral inflammations and dysbiotic biofilms [6].

Therefore, therapeutic agents for oral mucositis should ideally possess moisturizing, anti-inflammatory, and antibacterial properties.

Currently, treatment options for oral mucositis mainly include mouthwashes and topical corticosteroids. However, because these agents are typically administered as liquids or ointments, their retention in the oral cavity is limited owing to continuous salivary flow. Consequently, sustained drug-release properties are desired to achieve prolonged therapeutic effects. Freeze-dried hyaluronic acid (HA) sheets (hereafter referred to as HA sheets) are promising candidates because they rapidly transition to a gel-like structure and have the ability to remain in place for several hours [7]. A previous study using a hamster model of oral mucositis showed that the application of HA sheets promoted early healing [8]. However, few studies have investigated the incorporation of antibacterial components into HA sheets and their release behaviors.

Persimmons (*Diospyros kaki*) are edible fruits, and extracts obtained from astringent persimmons have strong protein-modulating and antibacterial activities [9]. Persimmon juice (kakishibu) is rich in polyphenols, including tannins. Owing to their numerous phenolic hydroxyl groups, persimmon tannins are reported to exert potent antioxidant, immunomodulatory, antibacterial, and antiviral effects [10,11,12,13]. Persimmon tannins have been reported to exert antibacterial activity against oral polymicrobial biofilms; for example, PS-M reduced colony-forming units in salivary biofilms in a concentration-dependent manner, with 4.0 wt% PS-M showing an effect comparable to that of 0.2 wt% chlorhexidine [14]. Therefore, persimmon tannins may be effective against oral bacterial communities, including representative facultative and anaerobic bacteria. In addition, tannins, such as tannic acid, have been reported to inhibit *Candida* species, including *Candida albicans* [15]. Persimmon-derived products have a long history of use as foods and traditional remedies, and persimmon tannins have been reported to exhibit diverse biological activities with generally low documented toxicity [16,17]. These characteristics support their potential use as antibacterial components in HA sheets. Nevertheless, the cytocompatibility of persimmon tannin-containing HA sheets should be evaluated before their application to the oral mucosa.

In this study, we developed freeze-dried HA sheets containing persimmon tannin and evaluated their potential as topical materials for cancer treatment–related oral mucositis. The release profile of persimmon tannin and the pH of the surrounding medium were examined over time. Antimicrobial activity was assessed against representative oral microorganisms, including *Streptococcus mitis*, *Prevotella intermedia*, and *Candida albicans*. In addition, the effects of HA sheets and antibacterial HA sheets on cell viability were evaluated using human oral cell line T3M-1 to assess their in vitro cytocompatibility.

## 2. Results

**Figure 1:**
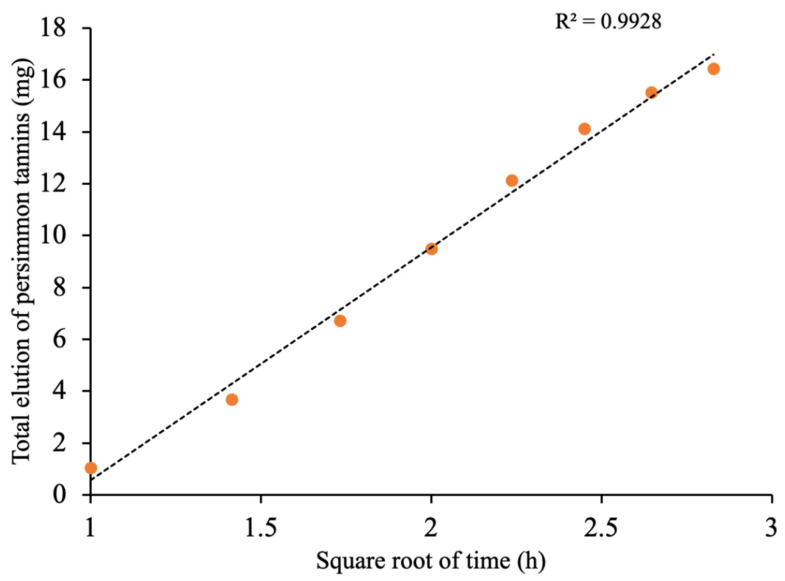
**Time-dependent cumulative amount of persimmon tannin released from antibacterial HA sheet.** The cumulative amount of persimmon tannin released into PBS was measured up to 8 h using the Folin–Ciocalteu method. Data are presented as mean ± standard deviation (*n* = 3).

During the release test, the amount of persimmon tannin released over time was measured. Each antibacterial HA sheet contained 80 mg of PS-M, corresponding to 17.2 mg of persimmon tannin. One hour after the start of the release test, 1.03 mg of persimmon tannin had been released. The cumulative amount of persimmon tannin increased with time and showed a linear relationship with the square root of time (R^2^ = 0.9928), reaching a cumulative release of 14.12 mg at 6 h and 16.41 mg at 8 h. Thus, approximately 95% of the persimmon tannin contained in the antibacterial HA sheet was released within 8 h (Figure 1).

**Figure 2:**
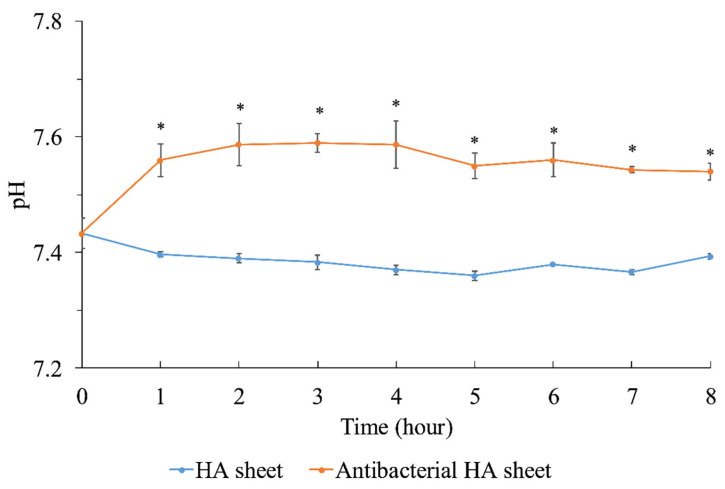
**Time-dependent changes in pH of eluates from HA sheets and antibacterial HA sheets.** HA sheets and antibacterial HA sheets containing persimmon tannin were immersed in PBS, and the pH of the surrounding PBS was measured every hour for up to 8 h without PBS replacement. Data are presented as mean ± standard deviation (*n* = 3). * *p* < 0.05 versus HA sheet at the same time point.

The pH of the surrounding phosphate-buffered saline (PBS) solution was monitored for 8 h. The mean pH in the HA sheet group ranged from 7.36 to 7.43, whereas that in the antibacterial HA sheet group ranged from 7.43 to 7.59. Although the difference was small, the pH was significantly higher in the antibacterial HA sheet group than in the HA sheet group from 1 to 8 h (Figure 2).

**Figure 3:**
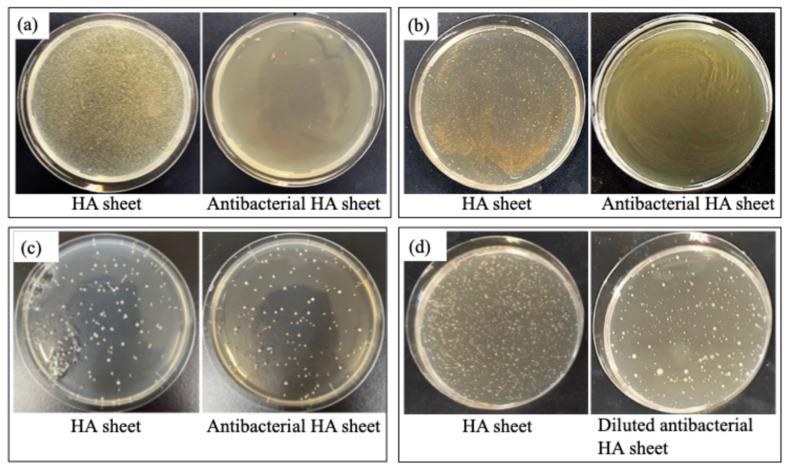
**Representative colony formation after incubation with HA sheet, antibacterial HA sheet, and diluted antibacterial HA samples.** Photographs of colonies recovered from (**a**) *Streptococcus mitis*, (**b**) *Prevotella intermedia*, and (**c**) *Candida albicans* after 6 h incubation on HA sheets or antibacterial HA sheets. Panel (**d**) shows colony formation of *S. mitis* after 1 h incubation with diluted HA and diluted antibacterial HA samples, which was performed to evaluate whether antibacterial activity was retained under a lower-concentration condition. Left panels show colonies recovered from HA sheet or diluted HA control, and right panels show colonies recovered from antibacterial HA sheet or diluted antibacterial HA sample. The images shown are representative of three independent experiments. (*n* = 3).

**Figure 4:**
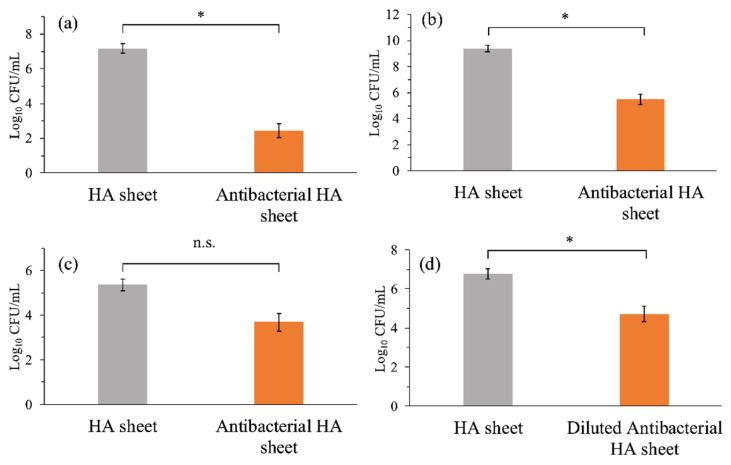
**Viable counts of microorganisms after incubation with HA sheet, antibacterial HA sheet, and diluted antibacterial HA sheet.** Viable counts are shown as log_10_ colony-forming units (CFU)/mL. Panels (**a**–**c**) show viable counts of (**a**) *S. mitis*, (**b**) *P. intermedia*, and (**c**) *C. albicans* after 6 h incubation on HA sheets or antibacterial HA sheets. Panel (**d**) shows viable counts of *S. mitis* after 1 h incubation with diluted HA and diluted antibacterial HA samples to assess antibacterial activity under a lower-concentration condition. Data are presented as mean ± standard deviation from three independent experiments. * *p* < 0.05 versus the corresponding HA control; n.s., not significant.

In the antibacterial assay, colony counts after incubation were compared between the HA sheet and antibacterial HA sheet groups. Compared with the HA sheet group, the antibacterial HA sheets markedly reduced colony counts of *Streptococcus mitis* and *Prevotella intermedia*, whereas no reduction was observed for *Candida albicans* (Figure 3 and Figure 4). Specifically, for *S. mitis*, the bacterial count after 6 h incubation was 2.8 × 10^7^ CFU/mL with HA sheets and 8.4 × 10^2^ CFU/mL with antibacterial HA sheets. For *P. intermedia*, the bacterial count was 2.9 × 10^9^ CFU/mL with HA sheets and 4.3 × 10^5^ CFU/mL with antibacterial HA sheets. In contrast, for *C. albicans*, the counts were 1.4 × 10^5^ CFU/mL with HA sheets and 2.3 × 10^5^ CFU/mL with antibacterial HA sheets, indicating no reduction in fungal counts (Figure 3 and Figure 4).

To determine whether antibacterial activity was retained at lower concentrations, an additional 1 h assay was performed using diluted samples. In this assay, the *S. mitis* count was 7.33 × 10^6^ CFU/mL in the diluted HA control and 7.27 × 10^4^ CFU/mL with the diluted antibacterial HA sample. The antibacterial activity (R) was 2.0, indicating that the diluted antibacterial HA sample retained antibacterial activity against *S. mitis* after 1 h of incubation (Figure 3d and Figure 4d).

**Figure 5:**
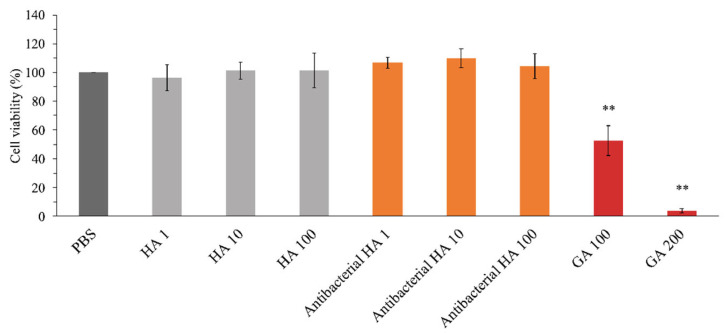
**Effects of HA sheets and antibacterial HA sheets on T3M-1 cell viability.** T3M-1 cells were exposed for 24 h to HA sheet or antibacterial HA sheet solutions at final concentrations of 1, 10, and 100 µg/mL. Glyceraldehyde (GA), used as a positive control, was tested at final concentrations of 100 and 200 µg/mL. Cell viability was determined using the CellTiter-Glo^®^ Luminescent Cell Viability Assay and expressed as a percentage relative to the PBS-treated control. Data are presented as the mean ± standard deviation of three independent experiments, each performed in triplicate. ** *p* < 0.01 versus the PBS-treated control, one-way ANOVA followed by Tukey’s honestly significant difference test.

The effects of HA sheets and antibacterial HA sheets on T3M-1 cell viability were evaluated after 24 h of exposure. Neither HA sheet nor antibacterial HA sheet solutions at concentrations of 1, 10, or 100 µg/mL significantly reduced cell viability compared with the PBS-treated control. Cell viability remained between 96.4% and 109.9% across these treatment groups. In contrast, glyceraldehyde, used as a positive control, significantly reduced cell viability to 52.4 ± 10.4% at 100 µg/mL and 3.9 ± 1.5% at 200 µg/mL (Figure 5). These results indicate that the antibacterial HA sheet solution did not exhibit detectable cytotoxicity toward T3M-1 cells at concentrations up to 100 µg/mL under the tested conditions.

## 3. Discussion

Anticancer drugs and radiotherapy used in cancer treatment also affect normal cells. In particular, basal cells of the oral mucosa, which have high proliferative activity, are susceptible to direct damage by anticancer drugs, leading to apoptosis and the development of oral mucositis [18,19]. Cancer treatment–related oral mucositis can be classified into five stages: (1) the initiation stage, in which damage occurs to basal cells; (2) and (3) the message generation and amplification stages, during which the epithelium is shed; (4) the ulceration stage, characterized by ulcer formation; and (5) the healing stage [20,21].

Clinical manifestations of oral mucositis include pain, bleeding, and discomfort during eating. These symptoms typically appear approximately 7 days after the start of treatment and peak around days 10–14 [22]. Freeze-dried HA sheets are expected to be particularly useful during the ulceration stage of oral mucositis when epithelial breakdown leads to exposed ulcers, severe pain, and marked dryness. At this stage, ulcers can serve as portals of entry for oral microorganisms; therefore, adding antibacterial activity is critical to reduce bacterial burden and the risk of secondary infection [23,24]. HA sheets may also contribute to the subsequent healing stage by maintaining hydration and providing a protective barrier that supports mucosal recovery [8]. Accordingly, the sustained release profile of persimmon tannin from the antibacterial HA sheets observed in this study suggests that the sheets can provide time-limited local antibacterial coverage, particularly during overnight use, when salivary clearance is reduced. However, because the ulcerative phase typically persists for several days, repeated applications are required to maintain coverage throughout the course of mucositis.

In the present study, experimental conditions were designed to approximate overnight use of antibacterial HA sheets. Salivary secretion decreases markedly during sleep; a typical resting salivary flow rate has been described to be as low as approximately 0.03 mL/min, corresponding to approximately 2 mL per hour [25]. In addition, only a small residual volume of saliva remains in the mouth immediately after swallowing (on average, ~0.8 mL), with reported values ranging roughly from ~0.6 to ~0.9 mL depending on the study population and methodology [26]. Therefore, antibacterial assays were conducted to evaluate whether the sheets could exert antimicrobial activity in a low-volume setting relevant to sleep. In the release test, the medium was refreshed in 2.5 mL aliquots as a practical approximation of a low-volume intraoral environment after swallowing, while ensuring stable contact between the sheets and the surrounding fluid for reproducible sampling. Although this in vitro setup cannot fully replicate the complex intraoral dynamics during sleep, it provides a controlled low-volume condition that approximates the salivary volume relevant to overnight use.

The causes of oral mucositis include direct cytotoxic effects of anticancer drugs, impaired immune function, the effects of radiation therapy, poor oral hygiene, malnutrition, and oral dryness [27]. Among these factors, decreased salivary flow due to anticancer drugs reduces the self-cleaning capacity of the oral cavity and has been reported to increase the risk of infection [28]. Hyaluronic acid is highly hydrophilic and has a strong water-holding capacity. Therefore, it can maintain mucosal hydration even when salivary flow is reduced. Moreover, HA has been reported in both in vitro and in vivo studies to promote wound healing and exert anti-inflammatory effects [8,29,30]. However, because HA does not possess antibacterial activity against oral bacteria, it is necessary to add antibacterial components to HA sheets to prevent secondary infection associated with oral mucositis.

In this study, we evaluated the in vitro antibacterial activity and release behavior of freeze-dried HA sheets incorporating persimmon tannin as an antibacterial component.

The release test showed that the cumulative persimmon tannin release from the antibacterial HA sheets increased linearly with the square root of time (R^2^ = 0.9928), consistent with diffusion-controlled release under the tested conditions, and continued for up to 8 h. It has been reported that water diffusion in HA gels is markedly slower than that in bulk water and that the hydrolytic degradation of HA is slow, particularly under neutral aqueous conditions [31,32]. Thus, the diffusion-controlled release behavior observed in the present study suggests that hydration via water diffusion into the HA layer and polymer chain relaxation, rather than hydrolysis, are the dominant processes. Although HA sheets without persimmon tannin were not evaluated in this study, the effect of persimmon tannin incorporation on the dissolution behavior of the HA sheets could not be directly assessed. Nevertheless, the results suggest that antibacterial HA sheets may enable sustained intraoral delivery, with the release profile potentially influenced by salivary flow and mechanical stress in the oral cavity.

Because local pH can influence microbial growth and the chemical behavior of plant-derived phenolic compounds, the pH of the surrounding PBS was monitored during incubation. Friedman and Jürgens reported that the stability of several plant phenolic compounds is pH-dependent [33]. In the present study, pH remained within a narrow near-neutral to mildly alkaline range in both groups. Although the pH was significantly higher in the antibacterial HA sheet group than in the HA sheet group from 1 to 8 h, the difference was minimal. This slight increase may be attributed, at least in part, to the sodium carbonate contained in PS-M [14]. Importantly, the antibacterial HA sheets did not induce acidic conditions or marked alkalinization during the observation period. Therefore, the marked reductions in viable counts of *S. mitis* and *P. intermedia* are unlikely to be explained by pH changes alone and are attributed primarily to the antibacterial activity of released persimmon tannin.

In the antibacterial assay, antibacterial HA sheets markedly reduced viable counts of *S. mitis* and *P. intermedia*, whereas no clear inhibitory effect was observed against *C. albicans*. In the additional 1 h assay, antibacterial HA sheets were gelled in 28 mL of culture medium, and a 2 mL portion of each gel was used to evaluate whether antibacterial activity could be retained under diluted conditions. The diluted antibacterial HA sample reduced the viable count of *S. mitis* from 7.33 × 10^6^ to 7.27 × 10^4^ CFU/mL compared with the diluted HA control, corresponding to an antibacterial activity value of R = 2.0. This finding indicates that the antibacterial HA sheet retained antibacterial activity even after dilution.

The amount of persimmon tannin contained in the diluted sample was approximately 1.2 mg, which was comparable to the amount released from the antibacterial HA sheet during the first hour of the release test. In addition, persimmon tannin continued to be released thereafter. These findings suggest that antibacterial HA sheets may begin to exert antibacterial activity against oral bacteria from the early phase after application and maintain activity during sustained release.

The pronounced reduction in viable counts of *S. mitis* and *P. intermedia* observed in this study can be attributed to the multifaceted antibacterial mechanisms of persimmon tannins. First, because persimmon tannins have abundant phenolic hydroxyl groups, they can strongly bind to proteins and induce aggregation and crosslinking, thereby potentially impairing cell surface proteins and enzymes and reducing bacterial growth capacity [34]. Second, persimmon tannins may interact with membrane lipids and proteins, increasing membrane permeability and inducing metabolic disturbances and osmotic stress, which in turn lead to a rapid decrease in viable cell counts [16]. In addition, tannins, including persimmon tannins, have been reported to inhibit biofilm formation and the expression of quorum-sensing-related factors [35], suggesting interference with bacterial adhesion and colonization on mucosal surfaces. Collectively, the observed decrease in viable counts of *S. mitis* and *P. intermedia* likely reflects the combined effects of such membrane- and protein-targeted rapid antibacterial actions and the release behavior of persimmon tannin from the antibacterial HA sheets.

In contrast, the lack of a clear effect against *C. albicans* suggests that differences in the composition of the fungal cell wall and cell membrane compared with bacteria may limit the ability of persimmon tannin alone to induce lethal damage in fungi [36]. Therefore, effective control of fungal infections may require strategies such as combination with antifungal agents or optimization of the exposure concentration and duration of persimmon tannins.

The release profile of persimmon tannin obtained in this study, together with the retention characteristics of HA sheets, appears complementary for enabling prolonged, localized delivery of active components to sites of oral mucositis. In patients undergoing chemotherapy or radiotherapy, changes in the oral microbiota associated with reduced salivary secretion and impaired immune function contribute to secondary infections and delayed healing [37,38]. Therefore, antibacterial HA sheets may help prevent exacerbation of oral mucositis by combining moisturizing effects with suppression of the growth of oral commensal bacteria.

The concentration of PS-M used in the present study was selected with consideration for future intraoral application. Increasing the amount of PS-M may improve antibacterial efficacy; however, excessive amounts of persimmon tannin may increase astringency and reduce patient acceptance. Optimization of the PS-M concentration will therefore be an important subject for future investigation.

Cytocompatibility is an important consideration for the potential application of antibacterial HA sheets to ulcerated oral mucosa. In the present study, neither HA sheet nor antibacterial HA sheet solutions significantly reduced T3M-1 cell viability after 24 h of exposure at concentrations ranging from 1 to 100 µg/mL. In contrast, glyceraldehyde markedly reduced cell viability, confirming that the assay was capable of detecting cytotoxic effects. These findings indicate that the incorporation of persimmon tannin into the HA sheets did not produce detectable cytotoxicity in the human oral cell line under the tested conditions. Comparable findings were obtained in HK-2 cells, which were included as an additional human epithelial cell model widely used for in vitro toxicity assessment (Figure S1). These supplementary findings support the absence of marked cytotoxicity under the tested conditions.

This study has several limitations. First, the experiments were conducted as a single-exposure in vitro evaluation over 6 h. The effects under actual intraoral conditions, where salivary components and mucins are present, as well as in polymicrobial or biofilm models, remain unclear. Second, the limited effect on *C. albicans* highlights the need to include components that specifically target fungi. Third, although the antibacterial HA sheet solutions did not reduce T3M-1 cell viability after 24 h of exposure at concentrations up to 100 µg/mL, the present assay represents only an initial in vitro cytocompatibility evaluation. The effects of direct sheet contact, higher local concentrations, repeated exposure, and prolonged exposure were not assessed. Before clinical application, it will also be necessary to evaluate palatability, mucosal irritation, sensitization, tooth staining, and the safety of repeated long-term use.

Future research should focus on the pharmacological characterization of antibacterial HA sheets. Determination of the minimum inhibitory and bactericidal concentrations of persimmon tannin, along with evaluation of biofilm removal and growth inhibition in biofilms formed under saliva and polymicrobial co-culture conditions, are important steps toward clinical application. In addition, because the release rate and retention time of HA sheets may vary with the molecular weight of HA, systematic evaluation across different formulations is warranted. Comprehensive in vivo evaluations using a hamster model of oral mucositis should assess ulcer area, re-epithelialization, inflammatory cytokine levels, pain-related behavior, microbiota composition, and local tissue compatibility.

Although the present cell viability assay showed no detectable cytotoxicity in T3M-1 cells at concentrations up to 100 µg/mL, the possibility of local toxicity associated with direct sheet contact, higher local concentrations, and repeated long-term application cannot be excluded. Therefore, further direct-contact, sensitization, and in vivo mucosal safety assessments are required before clinical application.

Freeze-dried HA sheets containing persimmon tannins exhibited in vitro antibacterial effects against oral commensal bacteria and a practical sustained-release profile, suggesting their potential as a topical adjunctive therapy for cancer treatment–related oral mucositis. Furthermore, their application may help alleviate discomfort and reduce the risk of secondary infections associated with oral dryness. However, additional studies are essential to address fungal control and to establish its efficacy and safety under actual clinical conditions.

## 4. Materials and Methods

### 4.1. Experimental Materials

**Figure 6:**
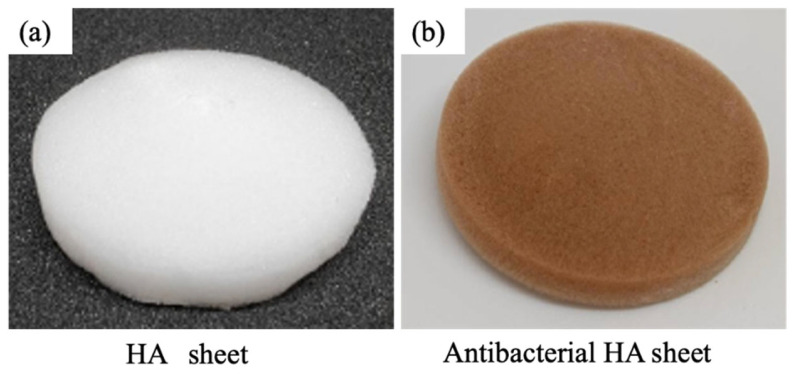
**Macroscopic appearance of the freeze-dried hyaluronic acid (HA) sheet and the antibacterial HA sheet containing persimmon tannin.** Representative photographs of (**a**) the HA sheet without persimmon tannin and (**b**) the antibacterial HA sheet incorporating persimmon tannin.

HA sheets were prepared by freeze-drying HA with a molecular weight of approximately 100 kDa according to previously reported methods [7,39]. The morphology, porous sheet structure, and physicochemical characteristics of the freeze-dried HA sheets have been characterized previously [39]. Antibacterial HA sheets were prepared by incorporating 80 mg of a commercial persimmon tannin product, PANCIL^®^ PS-M (hereafter referred to as PS-M; Rilis Co., Ltd., Osaka, Japan) into each HA sheet. The sheets were purchased from Ritapharma Co., Ltd. (Kyoto, Japan) and are shown in Figure 6. PS-M consists of 21.5% persimmon tannin, 66.4% trehalose, 6.6% sodium carbonate, and 4.5% water [14]. Accordingly, each antibacterial HA sheet contained approximately 17.2 mg of persimmon tannin.

In the antibacterial assays, *S. mitis* (ATCC 49456), *P. intermedia* (ATCC 25611), and *C. albicans* (ATCC 10231) were used. For *S. mitis*, trypticase soy broth (TSB; Becton, Dickinson and Co., Franklin Lakes, NJ, USA) supplemented with 3 mg/mL yeast extract (Biokar Diagnostic, Beauvais, France) was used as the culture medium. For *P. intermedia*, TSB supplemented with 5 mg/mL yeast extract, 0.5 mg/mL L-cysteine hydrochloride (FUJIFILM Wako Pure Chemical Co., Osaka, Japan), 5 μg/mL hemin (FUJIFILM Wako Pure Chemical Co.), and 1 μg/mL vitamin K (FUJIFILM Wako Pure Chemical Co.) was used. For *C. albicans*, yeast and mold (YM) medium (Becton, Dickinson and Co.) was used. Agar media were prepared by adding agar powder (Nacalai Tesque Inc., Kyoto, Japan) to each medium at a final concentration of 1.5%.

For the release test, PBS (FUJIFILM Wako Pure Chemical Co., Osaka, Japan) and Folin–Ciocalteu phenol reagent (Sigma-Aldrich, St. Louis, MO, USA) were used.

### 4.2. Release Test

**Figure 7:**
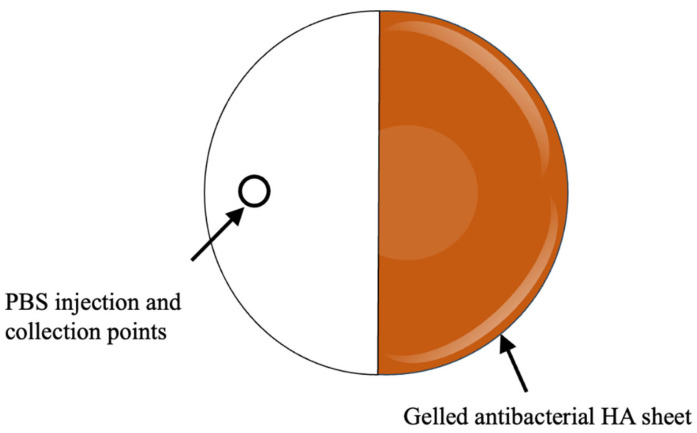
**Schematic diagram of the in vitro release test for persimmon tannin.** Gelled antibacterial HA sheet was placed on one side of a culture dish, and PBS was exchanged hourly through an inlet and an outlet to collect eluates for polyphenol quantification.

Antibacterial HA sheets were fully gelled by adding 2.5 mL of PBS (FUJIFILM Wako Pure Chemical Co.) and then spread over approximately half of the surface area of a 34 mm diameter culture dish (Corning Inc., Corning, NY, USA), as shown in Figure 7. On the side opposite, the antibacterial HA sheet, an inlet (PBS injection point) and an outlet (sample collection point) were set. At the inlet, 2.5 mL of PBS was added, and the dish was incubated at 37 °C with gentle shaking at 20 rpm. Every hour, 2.5 mL of PBS was collected from the outlet and replaced with 2.5 mL of fresh PBS. This procedure was repeated up to 8 h.

The total phenolic content in the collected PBS eluates was measured using the Folin–Ciocalteu method, with gallic acid as the standard, and expressed as persimmon tannin equivalents. Briefly, 1 mL of the collected solution was mixed with 2 mL of Folin–Ciocalteu reagent, followed by the addition of 2 mL of 10% Na_2_CO_3_ solution after 5 min. After incubation at room temperature in the dark for 1 h, absorbance was measured at 760 nm using a spectrophotometer (UV-1800; Shimadzu, Kyoto, Japan). The release test was independently performed in triplicate.

### 4.3. pH Measurement

HA sheets and antibacterial HA sheets were placed separately in 60 mm diameter culture dishes (Corning Inc.), and 6 mL of PBS (FUJIFILM Wako Pure Chemical Co.) was added to each dish. The samples were incubated at 37 °C with gentle shaking at 20 rpm. The pH of the surrounding PBS was measured every hour for up to 8 h without replacing the PBS during the measurement period. All experiments were performed in triplicate for each group. pH was measured using a LAQUA-PH-SE pH meter (Horiba Co., Ltd., Kyoto, Japan).

### 4.4. Antibacterial Assay

An appropriate liquid medium (2 mL) for each microorganism was added to the HA and antibacterial HA sheets to allow complete gelation. Microbial counts were determined and expressed as colony-forming units (CFU). Inoculum sizes were selected with reference to standard antimicrobial susceptibility testing methods and previous antifungal broth dilution assays, with modifications for the present gel sheet assay. Bacterial and fungal suspensions were adjusted to 1 × 10^7^ CFU/mL for *S. mitis* and *P. intermedia* and 1 × 10^4^ CFU/mL for *C. albicans* [40,41]. Subsequently, 100 μL of each suspension was inoculated onto the gelled samples, which were then incubated at 37 °C for 6 h under anaerobic conditions for *S. mitis* and *P. intermedia* and under aerobic conditions for *C. albicans*. Although *S. mitis* is a facultative anaerobe, it was incubated under anaerobic conditions in accordance with the cultivation guidance provided by the culture collection for this strain. Anaerobic conditions for both *S. mitis* and *P. intermedia* were generated using AnaeroPack-Anaero (Mitsubishi Gas Chemical Co., Inc., Tokyo, Japan) in a sealed anaerobic jar. After incubation, 8 mL of sterile distilled water was added to recover the microorganisms. A 1 mL aliquot of the recovered suspension was collected and serially diluted tenfold. One milliliter of an appropriate dilution was transferred to a sterile Petri dish and mixed with the corresponding molten agar medium using the pour-plate method [42]. Tryptic soy agar supplemented with yeast extract (3 mg/mL) was used for *S. mitis*, Brucella agar supplemented with hemin (10 μg/mL) and vitamin K (10 μg/mL) was used for *P. intermedia*, and YM agar was used for *C. albicans*. The plates were incubated at 37 °C for 48 h under the respective culture conditions. Plates containing countable numbers of colonies were selected, and viable microbial counts were calculated as CFU/mL based on the colony count, dilution factor, and plated volume.

To evaluate whether antibacterial activity was retained under lower concentration conditions approximating early-phase release, an additional diluted-sample assay was performed using *S. mitis*. In this assay, the antibacterial HA sheet was fully gelled in 28 mL of culture medium, and a 2 mL portion of the gel was transferred for testing. A 100 μL aliquot of bacterial suspension (1 × 10^7^ CFU/mL) was inoculated onto the 2 mL gel sample and incubated anaerobically for 1 h. Following the same recovery procedure, 1 mL of the suspension was recovered, mixed with agar medium, and incubated anaerobically at 37 °C for 48 h before colony counting.

Antibacterial activity (R) was calculated according to ISO 22196 using the following formula [43]:

R = log(A) − log(B)

where A is the viable count recovered from the HA sheet after 6 h of incubation, and B is the viable count recovered from the antibacterial HA sheet after 6 h of incubation. According to ISO 22196, an R value ≥ 2 was considered indicative of antibacterial activity.

### 4.5. Cell Viability Assay

T3M-1 Cl-10 cells (RCB1017) were obtained from the RIKEN BioResource Research Center, RIKEN Tsukuba Campus (Tsukuba, Japan). The cells were cultured in Ham’s F-10 medium (Sigma-Aldrich Co. LLC, St. Louis, MO, USA) supplemented with 10% fetal bovine serum (FBS; Biosera, Nuaillé, Maine-et-Loire, France) and penicillin–streptomycin solution (Sigma-Aldrich Co. LLC). Cells were maintained at 37 °C in a humidified atmosphere containing 5% CO_2_.

T3M-1 cells were seeded in 96-well microplates at a density of 3.2 × 10^4^ cells/cm^2^ (1.0 × 10^4^ cells/well) and incubated for 24 h. HA sheets and antibacterial HA sheets were dissolved in phosphate-buffered saline (PBS) and passed through a 0.22 µm membrane filter. The resulting solutions were diluted in culture medium and added to the cells at final concentrations of 1, 10, and 100 µg/mL. Glyceraldehyde (Nacalai Tesque) was used as a positive control at final concentrations of 100 and 200 µg/mL. Cells treated with the corresponding volume of PBS served as the vehicle control.

After 24 h of exposure, cell viability was assessed using the CellTiter-Glo^®^ Luminescent Cell Viability Assay (Promega Corporation, Madison, WI, USA) according to the manufacturer’s instructions. Luminescence was measured using a GloMax^®^ Navigator Microplate Luminometer (Promega Corporation). Cell viability was calculated as the luminescence of each treated group divided by the mean luminescence of the PBS-treated control group and expressed as a percentage. Each condition was tested in triplicate wells, and three independent experiments were performed.

### 4.6. Statistical Analysis

All experiments were independently performed in triplicate for each condition, and results are expressed as mean ± standard deviation. Statistical significance was evaluated using one-way analysis of variance (ANOVA), and multiple comparisons among groups were performed using Tukey’s honestly significant difference post hoc test. A *p* value < 0.05 was considered statistically significant.

## 5. Conclusions

Freeze-dried HA sheets containing persimmon tannin exhibited sustained release of persimmon tannin and significant in vitro antibacterial activity against *Streptococcus mitis* and *Prevotella intermedia*, whereas no inhibitory effect was observed against *Candida albicans* under the tested conditions. The sheets also showed no detectable cytotoxicity in T3M-1 cells at concentrations up to 100 µg/mL. These findings suggest that persimmon tannin-containing HA sheets may serve as a topical adjunct for reducing bacterial burden in cancer treatment–related oral mucositis. Further studies are required to establish antifungal efficacy, tissue compatibility, and safety under clinically relevant conditions.

## Data Availability

The datasets generated and analyzed in the current study are available from the corresponding authors upon reasonable request.

## Supplementary Materials

The following supporting information can be downloaded at: https://www.mdpi.com/article/10.3390/ijms27156880/s1.

## Abbreviations

The following abbreviations are used in this manuscript:

CFU	colony-forming units
HA	hyaluronic acid
PBS	phosphate-buffered saline
YM	yeast and mold

## References

[1] Naidu M.U.R., Ramana G.V., Rani P.U., Suman A., Roy P. (2004). Chemotherapy-induced and/or radiation therapy-induced oral mucositis-complicating the treatment of cancer. Neoplasia.

[2] Nishii M., Soutome S., Kawakita A., Yutori H., Iwata E., Akashi M., Hasegawa T., Kojima Y., Funahara M., Umeda M. (2020). Factors associated with severe oral mucositis and candidiasis in patients undergoing radiotherapy for oral and oropharyngeal carcinomas: A retrospective multicenter study of 326 patients. Support. Care Cancer.

[3] Lalla R.V., Latortue M.C., Hong C.H., Ariyawardana A., D’Amato-Palumbo S., Fischer D.J., Martof A., Nicolatou-Galitis O., Patton L.L., Elting L.S. (2010). A systematic review of oral fungal infections in patients receiving cancer therapy. Support. Care Cancer.

[4] Stoma I., Karpov I., Uss A., Rummo O., Milanovich N., Iskrov I. (2017). Diagnostic value of sepsis biomarkers in hematopoietic stem cell transplant recipients in a condition of high prevalence of gram-negative pathogens. Hematol./Oncol. Stem Cell Ther..

[5] Shelburne S.A., Sahasrabhojane P., Saldana M., Yao H., Su X., Horstmann N., Thompson E., Flores A.R. (2014). *Streptococcus mitis* strains causing severe clinical disease in cancer patients. Emerg. Infect. Dis..

[6] Könönen E., Fteita D., Gursoy U.K., Gursoy M. (2022). *Prevotella* species as oral residents and infectious agents with potential impact on systemic conditions. J. Oral Microbiol..

[7] Tanaka A., Nakano H., Yoneto K., Yoneto C., Furubayashi T., Suzuki K., Okae A., Ueno T., Sakane T. (2022). Topical xerostomia treatment with hyaluronate sheets containing pilocarpine. Biol. Pharm. Bull..

[8] Suzuki-Mishima K., Tanaka A., Kato-Kogoe N., Yamanegi K., Hirata A., Yoneto K., Yoneto C., Hamada W., Katsumi H., Furubayashi T. (2023). Topical Administration of Freeze-Dried Sheets of Hyaluronic Acid Promotes the Healing of Oral Mucositis in a Hamster Model. J. Hard Tissue Biol..

[9] Yamada Y., Yamamoto A.Y.A., Yoneda N., Nakatani N. (1999). Identification of Kaempferol from the Leaves of *Diospyros kaki* and its Antimicrobial Activity against *Streptococcus mutans*. Biocontrol Sci..

[10] Serrano J., Puupponen-Pimiä R., Dauer A., Aura A.M., Saura-Calixto F. (2009). Tannins: Current knowledge of food sources, intake, bioavailability and biological effects. Mol. Nutr. Food Res..

[11] Kitabatake M., Matsumura Y., Ouji-Sageshima N., Nishioka T., Hara A., Kayano S.-i., Ito T. (2021). Persimmon-derived tannin ameliorates the pathogenesis of ulcerative colitis in a murine model through inhibition of the inflammatory response and alteration of microbiota. Sci. Rep..

[12] Matsumura Y., Kitabatake M., Ouji-Sageshima N., Yasui S., Mochida N., Nakano R., Kasahara K., Tomoda K., Yano H., Kayano S.-i. (2017). Persimmon-derived tannin has bacteriostatic and anti-inflammatory activity in a murine model of *Mycobacterium avium complex* (MAC) disease. PLoS ONE.

[13] Ueda K., Kawabata R., Irie T., Nakai Y., Tohya Y., Sakaguchi T. (2013). Inactivation of pathogenic viruses by plant-derived tannins: Strong effects of extracts from persimmon (*Diospyros kaki*) on a broad range of viruses. PLoS ONE.

[14] Tomiyama K., Mukai Y., Saito M., Watanabe K., Kumada H., Nihei T., Hamada N., Teranaka T. (2016). Antibacterial action of a condensed tannin extracted from astringent persimmon as a component of food additive pancil PS-M on oral polymicrobial biofilms. BioMed. Res. Int..

[15] Moreira L.E.A., de Farias Cabral V.P., Rodrigues D.S., Barbosa A.D., Silveira M.J.C.B., Coutinho T.d.N.P., Barbosa S.A., Sá L.G.d.A.V., de Andrade Neto J.B., da Rocha S.N.C. (2024). Antifungal activity of tannic acid against *Candida* spp. and its mechanism of action. Braz. J. Microbiol..

[16] Wang R., Shi X., Li K., Bunker A., Li C. (2023). Activity and potential mechanisms of action of persimmon tannins according to their structures: A review. Int. J. Biol. Macromol..

[17] Bin Emran T., Amin M.A., Sutradhar B., Sweilam S.H., Shanmugarajan T.S., Arjun U., Durairaj A., Mahesh P.G., Jebastine M.I., Prasad P.D. (2026). A Comprehensive Review of the Phytochemical Constituents and Diverse Pharmacological Applications of *Diospyros kaki* (Persimmon) Across Traditional and Modern Medicine. Curr. Top. Med. Chem..

[18] Vera-Llonch M., Oster G., Ford C.M., Lu J., Sonis S. (2007). Oral mucositis and outcomes of allogeneic hematopoietic stem-cell transplantation in patients with hematologic malignancies. Support. Care Cancer.

[19] Cheng K.K.F., Leung S.F., Liang R.H.S., Tai J.W.M., Yeung R.M.W., Thompson D.R. (2009). A patient-reported outcome instrument to assess the impact of oropharyngeal mucositis on health-related quality of life: A longitudinal psychometric evaluation. Support. Care Cancer.

[20] Suhaimi N., Abdul Razak N.A.H., Ramli R. (2025). Pain Control in Oral Mucositis According to the Severity Scale: A Narrative Literature Review. J. Clin. Med..

[21] Sonis S.T. (2007). Pathobiology of oral mucositis: Novel insights and opportunities. J. Support. Oncol..

[22] Sonis S.T. (2009). Mucositis: The impact, biology and therapeutic opportunities of oral mucositis. Oral Oncol..

[23] Pulito C., Cristaudo A., Porta C., Zapperi S., Blandino G., Morrone A., Strano S. (2020). Oral mucositis: The hidden side of cancer therapy. J. Exp. Clin. Cancer Res..

[24] Min Z., Yang L., Hu Y., Huang R. (2023). Oral microbiota dysbiosis accelerates the development and onset of mucositis and oral ulcers. Front. Microbiol..

[25] Walsh L.J. (2007). Clinical aspects of salivary biology for the dental clinician. Int. Dent. S. Afr..

[26] Humphrey S.P., Williamson R.T. (2001). A review of saliva: Normal composition, flow, and function. J. Prosthet. Dent..

[27] Lalla R.V., Bowen J., Barasch A., Elting L., Epstein J., Keefe D.M., McGuire D.B., Migliorati C., Nicolatou-Galitis O., Peterson D.E. (2014). MASCC/ISOO clinical practice guidelines for the management of mucositis secondary to cancer therapy. Cancer.

[28] Jensen S.B., Pedersen A.M.L., Vissink A., Andersen E., Brown C.G., Davies A.N., Dutilh J., Fulton J.S., Jankovic L., Lopes N.N.F. (2010). A systematic review of salivary gland hypofunction and xerostomia induced by cancer therapies: Prevalence, severity and impact on quality of life. Support. Care Cancer.

[29] Chen W.Y., Abatangelo G. (1999). Functions of hyaluronan in wound repair. Wound Repair Regen..

[30] Kuppa S.S., Kang J.Y., Yang H.Y., Lee S.C., Sankaranarayanan J., Kim H.K., Seon J.K. (2024). Hyaluronic acid viscosupplement modulates inflammatory mediators in chondrocyte and macrophage coculture via MAPK and NF-κB signaling pathways. ACS Omega.

[31] Tokita Y., Okamoto A. (1995). Hydrolytic degradation of hyaluronic acid. Polym. Degrad. Stab..

[32] Kruk D., Rochowski P., Masiewicz E., Wilczynski S., Wojciechowski M., Broche L.M., Lurie D.J. (2019). Mechanism of Water Dynamics in Hyaluronic Dermal Fillers Revealed by Nuclear Magnetic Resonance Relaxometry. Chemphyschem.

[33] Friedman M., Jürgens H.S. (2000). Effect of pH on the stability of plant phenolic compounds. J. Agric. Food Chem..

[34] Liu M., Feng M., Yang K., Cao Y., Zhang J., Xu J., Hernández S.H., Wei X., Fan M. (2020). Transcriptomic and metabolomic analyses reveal antibacterial mechanism of astringent persimmon tannin against Methicillin-resistant *Staphylococcus aureus* isolated from pork. Food Chem..

[35] Farha A.K., Yang Q.-Q., Kim G., Li H.-B., Zhu F., Liu H.-Y., Gan R.-Y., Corke H. (2020). Tannins as an alternative to antibiotics. Food Biosci..

[36] Gow N.A.R., Latge J.-P., Munro C.A. (2017). The fungal cell wall: Structure, biosynthesis, and function. Microbiol. Spectr..

[37] Ue A., Tamaki Y., Usuda H., Yamamori U., Burioka H., Nagano N., Uno M., Sonoyama Y., Okamoto T., Wada K. (2025). An increase in *Fusobacterium* is associated with the severity of oral mucositis after radiotherapy. Sci. Rep..

[38] Vistoso Monreal A., Polonsky G., Shiboski C., Sankar V., Villa A. (2022). Salivary Gland Dysfunction Secondary to Cancer Treatment. Front. Oral Health.

[39] Tanaka A., Takata T., Katsumi H., Sawai Y., Nakano H., Yoneto C., Yoneto K., Furubayashi T., Sakane T. (2026). Development of Freeze-Dried Hyaluronic Acid Sheets for Healing Oral Mucositis: Influence of Hyaluronic Acid Molecular Weight and Nicotinamide Mononucleotide Loading on Healing Efficacy. J. Funct. Biomater..

[40] Clinical and Laboratory Standards Institute (2018). Methods for Antimicrobial Susceptibility Testing of Anaerobic Bacteria.

[41] Lukáč M., Slobodníková L., Mrva M., Dušeková A., Garajová M., Kello M., Šebová D., Pisárčik M., Kojnok M., Vrták A. (2024). Caffeic Acid Phosphanium Derivatives: Potential Selective Antitumor, Antimicrobial and Antiprotozoal Agents. Int. J. Mol. Sci..

[42] U.S. Food and Drug Administration BAM Chapter 3: Aerobic Plate Count.

[43] (2011). Measurement of Antibacterial Activity on Plastics and Other Non-Porous Surfaces.

