# Supplementary material for: Antimicrobial Properties of Freeze-Dried Hyaluronic Acid Sheets Containing Persimmon Tannin
Source: Int J Mol Sci. 2026 Aug 1;27(15):6880. doi: 10.3390/ijms27156880 (PMC13467313; doi:10.3390/ijms27156880)
Supplement: Supplementary file 1 [file ijms-27-06880-s001.zip › ijms-4442530-supplementary.pdf]

Supplementary Materials

Cell culture and viability assay using HK-2 cells

HK-2 cells (CRL-2190) were obtained from the American Type Culture Collection (ATCC, Manassas, VA, USA). The cells were cultured in Dulbecco's modified Eagle's medium/Nutrient Mixture F-12 Ham (DMEM/F-12 Ham; Sigma-Aldrich Co. LLC) supplemented with 10% fetal bovine serum (Biosera) and penicillin–streptomycin solution (Sigma-Aldrich Co. LLC). Cells were maintained at 37°C in a humidified atmosphere containing 5% CO_2.

HK-2 cells were seeded in 96-well microplates at a density of 3.2×10^4 cells/cm^2 (1.0×10^4 cells/well) and incubated for 24 h. HA sheet and antibacterial HA sheet solutions were prepared and applied at final concentrations of 1, 10, and 100 μg/mL using the same procedure described for T3M-1 cells. Glyceraldehyde was used as a positive control at final concentrations of 200 and 400 μg/mL. After 24 h of exposure, cell viability was measured using the CellTiter-Glo® Luminescent Cell Viability Assay and expressed relative to the PBS-treated control. Each condition was tested in triplicate wells, and three independent experiments were performed.

Supplementary Results

Neither the HA sheet nor the antibacterial HA sheet solutions significantly reduced HK-2 cell viability at concentrations of 1–100 μg/mL compared with the PBS-treated control. Glyceraldehyde significantly reduced cell viability at 200 and 400 μg/mL.

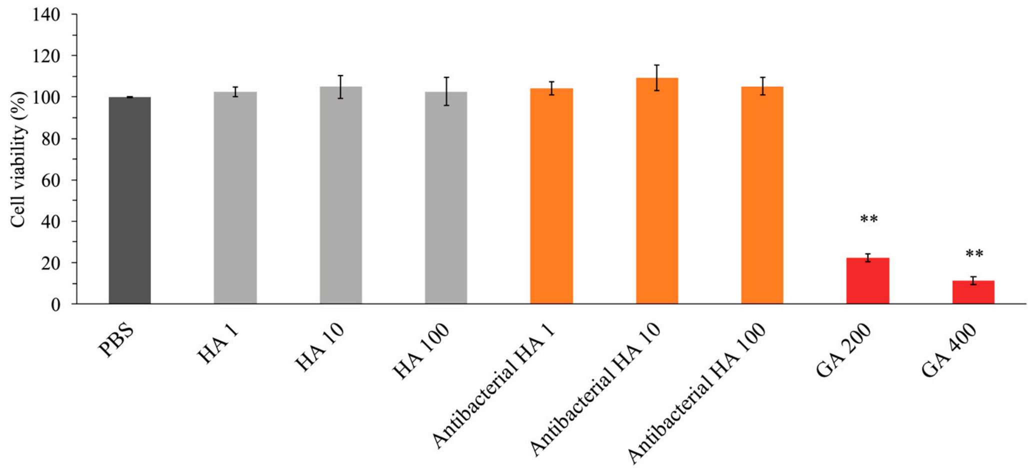

Figure S1. Effects of HA sheets and antibacterial HA sheets on HK-2 cell viability. HK-2 cells were exposed for 24 h to HA sheet or antibacterial HA sheet solutions at final concentrations of 1, 10, and 100 μg/mL. Glyceraldehyde, used as a positive control, was tested at final concentrations of 200 and 400 μg/mL. Cell viability was expressed relative to the PBS-treated control. Data are presented as the mean ± standard deviation of three independent experiments, each performed in triplicate. **$p < 0.01$ versus the PBS-treated control, one-way ANOVA followed by Tukey's honestly significant difference test.
